# Single-Port Laparoscopic Surgery for Inflammatory Bowel Disease

**DOI:** 10.1155/2012/106878

**Published:** 2012-04-29

**Authors:** Emile Rijcken, Rudolf Mennigen, Norbert Senninger, Matthias Bruewer

**Affiliations:** Department of General and Visceral Surgery, Muenster University Hospital, Waldeyerstraße 1, 48149 Muenster, Germany

## Abstract

*Background*. Single Port Laparoscopic Surgery (SPLS) is being increasingly employed in colorectal surgery for benign and malignant diseases. The particular role for SPLS in inflammatory bowel disease (IBD) has not been determined yet. In this review article we summarize technical aspects and short term results of SPLS resections in patients with Crohn's disease or ulcerative colitis. *Methods*. A systematic review of the literature until January 2012 was performed. Publications were assessed for operative techniques, equipment, surgical results, hospital stay, and readmissions. *Results*. 34 articles, published between 2010 and 2012, were identified reporting on 301 patients with IBD that underwent surgical treatment in SPLS technique. Surgical procedures included ileocolic resections, sigmoid resections, colectomies with end ileostomy or ileorectal anastomosis, and restorative proctocolectomies with ileum-pouch reconstruction. There was a wide variety in the surgical technique and the employed equipment. The overall complication profile was similar to reports on standard laparoscopic surgery in IBD. *Conclusions*. In experienced hands, single port laparoscopic surgery appears to be feasible and safe for the surgical treatment of selected patients with IBD. However, evidence from prospective randomized trials is required in order to clarify whether there is a further benefit apart from the avoidance of additional trocar incisions.

## 1. Introduction

Single-Port Laparoscopic Surgery (SPLS) is a development in the field of minimally invasive surgery that aims to minimize the surgical access trauma by reducing the number of abdominal incisions to a single incision. The specimen can be extracted via the incision for the single port. Advocates of SPLS claimed potential advantages for this approach when compared to standard multitrocar laparoscopic surgery, such as better cosmetic results, decreased postoperative pain, or faster recovery, but proof for this is lacking. SPLS has been shown to be feasible in colorectal surgery in a rapidly increasing number of publications [1–4]. Various procedures in colonic surgery have been performed in SPLS technique: Both right and left hemicolectomies, sigmoid resections, and proctocolectomies with formation of an ileum-J-pouch have been reported (review in [5–7]). In these studies, indications for SPLS colonic operations included chronic diverticulitis, Crohn's disease, ulcerative colitis, familial adenomatous polyposis, large adenoma, and carcinoma of the colon. Most of these reports were limited to small patient series, demonstrating the technical feasibility of the SPLS procedure. In contrast, comparative studies of the SPLS technique with traditional laparoscopic or open surgery in larger series of patients are rare. Therefore, the true value of the SPLS technique in colonic surgery remains unclear at present. Nevertheless, the SPLS-technique might be interesting, especially in patients with benign disorders such as inflammatory bowel disease (IBD). However, the surgical treatment of patients with IBD remains challenging, since many patients present with fistulizing disease, abscesses, cachexia, recurrent disease, and compromised healing capacity following the application of immunosuppressive drugs. The aim of this systematic review was to analyze the currently available literature on single-port laparoscopic surgery in patients with IBD with respect to feasibility, reported techniques, and safety and to identify potential benefits of this new technique in this particular group of patients.

## 2. Methods

### 2.1. Article Identification and Selection

A systematic query was performed using the data bases Pubmed, Medline, and Web of Science. Articles published from January 2000 until January 2012 were considered. Search terms included “single-port laparoscopic surgery,” “colorectal surgery,” “single access,” “single incision,” “SPLS;” “SAS,” “SPA;” “SILS,” “LESS,” “MISS,” “SILC,” “OPUS,” “SIMPLE,” “colon,” “bowel,” “small bowel,” “Crohn's disease,” “ulcerative colitis,” and “IBD”. There was no language restriction. Original articles, case reports, and technical notes were considered, whereas experimental studies in animal models, review articles, editorials, abstracts, and congress reports were excluded. Studies combining SPLS with other access routes or using a robotic approach were also excluded. Studies reporting SPLS in colorectal surgery in other conditions than IBD were excluded. Publications describing SPLS in a mixed cohort undergoing small or large bowel surgery were considered only for the reported IBD patients, whereas those patients with appendicitis, benign large, or small bowel conditions other than IBD, or with malignant colorectal disease were excluded from analysis.

### 2.2. Article Analysis

Data were extracted by one surgeon, experienced in both single-port and standard laparoscopic colorectal surgery. Suitable articles were divided into different study types such as case reports, case series, or case-controlled studies. The studies were assessed for the following criteria: indication, SPLS-procedure, SPLS-port used, SPLS-port position, incision length, specimen extraction site, technical equipment, previous abdominal surgery, operation time, conversions, complications, wound infections, length of hospital stay, reoperations, and readmissions.

## 3. Results

### 3.1. Study Retrieval

**Figure 1:**
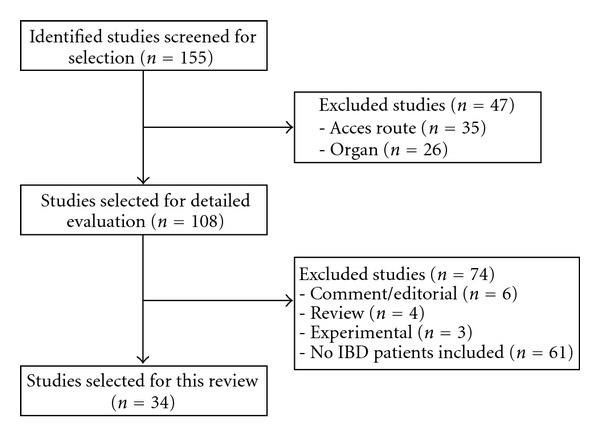
Single-Port Laparoscopic Surgery for inflammatory bowel disease: selection of analyzed studies.

The primary search found 155 potentially relevant studies. After eliminating studies in which the access route to the abdomen was not per SPLS or the organ studied was not small or large bowel, 108 studies remained. Of these, 34 studies reported on SPLS in patients with IBD (Figure 1). These 34 studies met the inclusion criteria and were analyzed in detail. The selected studies were comprised of 5 case reports, 19 case series, and 10 case-controlled studies. There were no prospectively randomized studies available.

The 34 selected studies reported on 1023 SPLS patients in total, including 301 patients with IBD. Among these, there were 150 patients with Crohn's disease and 151 patients with ulcerative colitis. 8 studies described data of 10 or more IBD patients. However, since 5 groups of surgeons contributed more than one (2–4) publication to the final selection, quite a number of individuals might have been repeatedly reported, substantially reducing the actual number of reported IBD patients treated by SPLS technique. In contrast, 19 studies originated from researchers with only one publication on SPLS including IBD patients. 14 studies were restricted to SPLS in IBD patients only, whereas the other 20 studies included IBD patients in a mixed cohort of SPLS colorectal surgery. Among the 14 IBD-only studies, there were 5 case reports, 6 case series including more than one IBD patient, and 3 case-controlled studies. The selected studies were published in the years 2010 (*n* = 8) and 2011 (*n* = 21), and 2012 (*n* = 5), including those studies that were published online ahead of print.

### 3.2. Surgical Technique and Procedures

The reported SPLS procedures in IBD patients included 117 ileocolic resections (ileocecal resection, right hemicolectomy, and ileocolic resection for recurrent Crohn's disease), 13 sigmoid resections, 3 left hemicolectomies, 77 subtotal colectomies with end ileostomy, 3 colectomies with ileorectal anastomosis, and 52 restorative proctocolectomies with ileum-pouch reconstruction (Tables 1–3). Furthermore, SPLS small bowel resections and stricturoplasties for Crohn's disease were reported. Several studies that report on SPLS colorectal surgery in larger mixed cohorts did not specify whether the single procedures were performed in patients with IBD or in patients with other specific diagnoses [8–13]. 20 studies were restricted to a single type of resection, whereas 14 studies reported more than one kind of resection. 31 studies specified the type of port applied, of which 7 studies reported 2–4 different types of ports applied in their particular series. Applied SPLS-ports were SILS (Covidien, Norwalk, CT) in 20 studies, Triport (Olympus, Southend, UK and Advanced Surgical Concepts, Wicklow, Ireland) in 7 studies, Quadport (Olympus America, Center Valley, PA and Advanced Surgical Concepts, Wicklow, Ireland) in 3 studies, GelPort respectively GelPoint (Applied Medical, Rancho Santa Margarita, CA) in 11 studies, SSL (Ethicon Endosurgery, Cincinnati, OH) in 4 studies, and Spider surgical system (Transenterix, Durham, NC) in 1 study. 1 study inserted 3 trocars trough a single incision tightened by a purse string [14], whereas other authors placed multiple trocars through the fascia separately trough a single skin incision secured by soft tissue flaps [4, 10]. 14 studies reported the use of one or more additional trocars apart from the single port in some cases when difficulties occurred intraoperatively. The umbilicus was the most frequent site of abdominal access in SPLS procedures (20/34). Three authors used a paraumbilical access in patients with Crohn's disease [12, 15, 16]. In IBD patients undergoing a procedure with the need for an ileostomy, such as colectomy, the ileostomy site was used for insertion of the SPLS-port in 15 studies. Other authors reported the use of the left iliac fossa as access site [17], whereas four authors also reported a suprapubic insertion site for the SPLS port [8, 9, 12, 14]. 31/34 studies reported extraction of the specimen using the SPLS-port site, which had to be enlarged in several cases. Three authors also reported transanal specimen delivery in some cases [18–20] and one study reported transvaginal extraction of the excised colon [21]. Another study reported specimen delivery in a scar located at McBurney's site in a case of enterocutaneous fistula [22]. In studies reporting right-sided resections, ileocolic anastomoses were performed extracorporeally in most cases (19/22) and intracorporeally in one, while the method was not specified in two studies. Reconstruction after left-sided colonic resection was performed transanally (17/20) using double stapling in the vast majority of studies and was only in rare cases handsewn. 24 of 34 studies reported the use of standard laparoscopy instruments for SPLS-procedures, whereas only three authors stated the use of specially adjusted curved SPLS instruments [9, 21, 23]. The optical systems used were flexible tip cameras in 7 studies, straight 5 mm 30° optics in 15 studies, straight 10 mm 30° optics in 9 studies, straight 5 mm 0° optic in two studies, and a straight 10 mm 0° optic in 1 study. 10 studies reported routine preoperative bowel preparation for SPLS colorectal procedures. 19 studies included patients with previous abdominal surgery in SPLS procedures.

### 3.3. Exclusion Criteria for SPLS Procedures in IBD

The vast majority of the SPLS procedures in IBD were selected cases in a nonemergency setting. 13 studies reported exclusion criteria for SPLS procedures in patients with IBD: these were in particular: body habitus, respectively, BMI > 36 kg/m² [11–13, 23–27], ASA-classification >3 [23], respectively, significant associated comorbidities [24, 25, 28], hemodynamic instability [27], extensive previous abdominal surgery [23–30], previous history of peritonitis [12, 13], emergency surgery such as colonic perforation and toxic megacolon [8, 12, 13, 23, 26, 28, 30], colonic dysplasia or malignancy [11, 26], respectively, low rectal malignancy [30], and pregnancy [29].

### 3.4. Technique of SPLS Right Hemicolectomy

22 studies described SPLS right hemicolectomies or ileocecal resections in patients with Crohn's disease (Table 1), including 4 case reports [8–17, 20–23, 27, 29, 31–36]. Most authors used the umbilicus for accessing the abdomen. The predominant technique was a medial-to-lateral approach with cephaled dissection of the mesentery to the duodenum with a thermal sealing device and/or an endoscopic stapler [9, 12, 23, 29, 30, 33, 36]. Subsequently, the ascending colon was mobilized past the right flexure. Other authors applied a posterior approach to mobilize the colon prior to mesenteric dissection [16, 35]. The ileum and the colon were transected either intra- [29] or extraperitoneally [9, 12, 16]. After extraction of the specimen at the SPLS port site, a side-to-side ileocolic anastomosis was performed using a stapling technique in an open extracorporeal fashion in the vast majority of the studies. Some authors created a loop ileostomy in cases of complicated Crohn's disease [34, 35].

### 3.5. Technique of SPLS Subtotal Colectomy

SPLS subtotal colectomies with terminal ileostomy in patients with IBD were reported in 14 studies (Table 2) [8, 11, 13, 17, 19, 20, 24–28, 30, 32, 37]. Two studies reported SPLS colectomy with ileorectal anastomosis [17, 30]. SPLS port insertion was usually accomplished at the previously marked ileostomy site [24, 25, 28, 37]. For SPLS colectomy, most authors commenced dissection at the right hemicolon, arguing this part to be the most difficult and associated with the highest risk for conversion, followed by further clockwise dissection [20, 24–26, 37]. Other authors, however, reported an early transsection of the distal sigmoid at the level of the promontory, followed by a distal to proximal dissection of the colon close to the bowel wall [28]. Dissection of the mesocolon was performed using sealing devices and endo-staplers were applied for transsection of the rectum in all selected studies. Extraction of the colon occurred at the ileostomy site followed by extracorporeal transsection of the terminal ileum, which was then turned into a terminal stoma after correct orientation of the small bowel.

### 3.6. Technique of SPLS Restorative Proctocolectomy

SPLS restorative proctocolectomies in patients with ulcerative colitis were reported in 12 studies [4, 8, 13, 17–20, 26, 27, 38–40]. In most of these, the SPLS port was inserted at the site chosen for the loop ileostomy in the right iliac fossa [18], while other studies reported insertion of the SPLS port at the umbilicus, using the ileostomy site or drain site for additional 5–12 mm ports in some cases [20, 38]. In patients with previous subtotal colectomy, SPLS was successfully performed using the stoma site after prior mobilization of the terminal stoma [18]. A medial to lateral approach was performed in most studies, and most authors began dissecting at the right hemicolon [18, 20, 38]. The entire colon was divided using sealing devices and divided at the level of the pelvic floor with an endo stapler in an anterior-posterior direction, introduced via the SPLS port. Extraction of the colon was carried out via the port site or transanally [18, 20]. The ileal J-pouch was constructed extracorporeally by linear staplers with a limb length of 15–20 cm and reinserted into the abdomen via the port site. Pouch-anal anastomosis was performed intracorporeally by double stapling [18, 38] or, in cases of proctomucosectomy, handsewn transanally [18, 20]. Virtually all authors reported a diverting loop ileostomy (Table 3).

### 3.7. Surgical Outcomes

Three main procedures in IBD were analyzed separately. Results from the literature for SPLS ileocecal resections and SPLS right hemicolectomies in Crohn's disease are depicted in Table 1. Results for SPLS subtotal colectomies for ulcerative colitis and Crohn's disease are shown in Table 2, and results for SPLS restorative proctocolectomies in ulcerative colitis are demonstrated in Table 3. It is noteworthy that authors reporting on mixed cohorts of different procedures in large series of patients often do not give data for specific procedures. Specific data were presented wherever possible and mixed data are indicated. Reported mean or median operation times for ileocolic resections varied from 77 to 155 min, for subtotal colectomy with end ileostomy from 112 to 206 min, and for reconstructive proctocolectomy with ileal pouch from 153 to 300 min. Reported median incision length was 35 (20–55) mm. Several authors reported widening the initial incision for extraction of the specimen in Crohn's disease patients with enlarged mesentery.

For all SPLS procedures in IBD, cases of conversions to multiport surgery were reported in 14 studies and cases of conversion to open surgery were reported in 10 studies. Reasons for conversions were medically related issues such as intraoperative bleeding [20], firm adhesions and previous surgery [12, 20, 27, 29], fistulizing disease (interenteric fistula, conglomerate tumors, or masses [8, 16, 20], friability of the inflamed mesentery [12], obesity [8, 30], or technically related aspects such as gas leak [30], instable port placement [17], inappropriate traction [8, 12, 29], difficulties in flexure mobilization [9], and time constraints [17].

Complications in SPLS procedures in IBD were reported in 22 studies. These complications included anastomotic leakage, bleeding, ileus, bowel obstruction, intraabdominal abscesses, wound infections, delayed thermal injury to bowel, peristomal emphysema, ejaculation dysfunction, acute urine retention, incisional hernia, stenoses, and cardiovascular, pulmonary, and thromboembolic events (Tables 1–3). Re-operations due to complications were stated in 8 studies. Mortality was reported in 4 studies [8, 12, 29, 36] and specified in 3 of them. One case of mortality was reported after substantial intraoperative bleeding during externalization of the colon for an extracorporeal anastomosis after right hemicolectomy [36]. Another case of mortality due to pulmonary embolism was found in one study, although it remains unclear whether this was a patient with IBD [29]. A third case of mortality due to cardiopulmonary failure was reported in a patient undergoing SPLS sigmoidectomy for complicated diverticulitis [8].

## 4. Discussion

The current review of the literature shows that single-port laparoscopic surgery has gained entrance into the surgical treatment of patients with inflammatory bowel disease. The number of publications on the subject is growing at a fast pace: whereas first case reports arose in 2010, larger case series from specialized centers are now available that demonstrate the feasibility of SPLS in IBD. Additionally, some comparative studies have been published lately, mostly comparing SPLS to historical cohorts of patients with traditional multiport laparoscopic surgery. Evidence from prospectively designed, randomized studies concerning SPLS in IBD is not presently available. Therefore, benefits of SPLS in IBD were not demonstrated so far. Most of the currently available studies on the application of SPLS in colorectal surgery which include IBD patients are not restricted to single procedures in single pathological conditions, but rather describe mixed cohorts. As a consequence, it is not yet possible to perform a proper meta-analysis in order to evaluate the techniques in detail. However, it appears that nearly all IBD-related procedures that can be performed by standard multiport laparoscopy have now been performed in single-port technique as well. Although this has mostly been done by specialized surgeons, it demonstrates the general feasibility of SPLS in IBD. The SPLS procedures include stricturoplasties, small bowel resections, ileocolic resections, sigmoid resections, subtotal colectomies with terminal ileostomies, and reconstructive proctocolectomies with ileal pouches. SPLS proctocolectomy for ulcerative colitis has been reported in minors, too [40]. However, from the available literature, it becomes apparent that most authors applied SPLS predominantly in selected patients, and therefore SPLS is currently still far from becoming a routine procedure in IBD patients. Emergency cases were excluded from SPLS in the vast majority of publications [16, 24–26, 30]. From a technical point of view, most authors favor regular laparoscopic instruments, although a special 5 mm optic with a flexible tip seems to be rewarding in SPLS colorectal procedures [8]. Most authors applied commercially available SPLS ports, which were inserted through the umbilicus, paraumbilically, at the ileostomy site, or suprapubically depending on the specific procedure and the surgeon's preference. SPLS was performed for IBD in patients with prior (limited) abdominal surgery, but also in patients with recurrent Crohn's disease [14, 34, 35] or enterocutaneous fistula and abscesses [22, 35]. SPLS—in experienced hands—may therefore be a feasible approach even in complex patients. Limitations of SPLS in IBD patients appear to be similar to those encountered in standard multitrocar laparoscopy. Reasons for conversions were stated as occurrence of intraoperative bleeding, bowel injury, firm adhesions, intraenteral fistula, and masses. These reasons were also stated in the literature for IBD patients undergoing conversion during standard laparoscopic resections [41–45]. In terms of patient safety, SPLS for IBD offers a risk profile similar to standard multitrocar laparoscopic surgery. Postoperative complications reported include anastomotic leakage, bleeding, bowel obstruction, and intraabdominal abscesses. These are typical complications of colorectal surgery in IBD as seen in both standard multitrocar laparoscopic and open surgery [46, 47]. In contrast, delayed thermal injury as reported in two studies indicates inappropriate instrument handling in SPLS. Wound infections at the site of the SPLS port were reported by several authors. A reduction of the frequency of wound infections by reducing the number of incisions using SPLS is not likely to occur. The incidence of late complications such as incisional hernia should be objectified in future studies on the long-term outcome of SPLS patients. Furthermore, IBD-specific long-term complications such as recurrence of stenoses in Crohn's disease or pouchitis in ulcerative colitis are not likely to be influenced by the technique used for access to the abdomen in the primary operation. A reduction of peritoneal adhesions and consecutive bowel obstruction was postulated to be achieved by SPLS, but there are no long-term studies available so far which confirm this hypothesis. Surgery in patients with IBD does not differ substantially from surgery for other conditions, but the patients undergoing these procedures are often complex and challenging due to a previous history of the disease, nutritional status, septic manifestations such as fistulas and abscesses, and/or immunosuppresive drugs. In the present review of the literature, no specific data on the patient's exposure to immunosuppressive drugs could be retrieved. Some of the selected studies, however, reported preoperative administration of azathioprine, steroids, or biologicals [8, 16, 24, 25, 28, 35, 37], indicating that the application of these drugs does not represent a contraindication for SPLS. In patients undergoing restorative proctocolectomy for medically refractory ulcerative colitis, a three-stage SPLS procedure was advocated when patients received more than 20 mg of prednisolone or anti-TNF-*α* agents such as infliximab or adalimumab [8]. In some studies, benefits of SPLS in colorectal procedures such as shorter hospital stays [11, 15], reduction of estimated blood loss [13], reduced time to flatus and bowel movement [9], or better cosmetic results [9] were claimed, but results from these studies appear to be limited by inhomogeneous cohorts, small sample size with low statistical power, or possible selection bias. A small randomized prospective study including 16 SPLS patients and 16 patients with standard laparoscopic surgery in colon cancer found no differences in terms of morbidity and operation time [48]. In the available literature on SPLS in IBD, potential benefits have yet to be demonstrated.

In conclusion, the present review of the literature shows the feasibility of SPLS in patients with IBD in selected cases. The patient selection however depends on the surgeon's experience and the patient's condition. Currently, the literature on SPLS techniques in IBD is shifting from case reports on single applications to reports on larger series. At present there are no technical standards for SPLS procedures in IBD. Evidence from prospectively randomized trials is required to clarify whether there is a true benefit compared to standard laparoscopic techniques.

## Figures and Tables

**Table 1:** Perioperative results of SPLS ileoc resection-right hemicolectomy for Crohn's disease: included studies. Crohn-specific data were given wherever possible.

Author, year	Study type	Total number of SPLS patients	Disease (*n*, all SPLS patients)	SPLS ileocecal resection-Right hemicolectomy (Crohn/ total)	Elective: emergency	Final incision length (cm)	Ileocolic anastomosis	Additional trocars (*n*/cases)	Conversion to open surgery	Operative time (min)	Mortality (*n*/cases)	Morbidity (*n*/cases)	Reoperations (*n*/cases)	Hospital stay (*d*)	Read missions (*n*/cases)
Adair et al., 2010 [29]	CC	17	CD: 1 Carcinoma: 11 Adenoma: 4 Other: 1	1/17	n.s.	3.8^∗§^	Extracorporeal	2/17*	0/17*	139 ± 29.7^∗, §^	1/17* (pulmonary embolism*)	4/17 (wound infection: 1, Ileus 2, delayed thermal bowel injury: 1)	n.s.	3.9 ± 3.7^§^	n.s.

Heeney et al., 2010 [31]	CR	1	CD: 1	1/1	1 : 0	2.5	Extracorporeal	0/1	0/1	86	0/1	0/1	0/1	n.s.	0/1

Kawahara et al., 2010 [14]	CR	1	CD: 1	1/1	1 : 0	4.0	Extracorporeal	0/1	0/1	130	0/1	0/1	0/1	10	n.s.

Keshava et al., 2010 [33]	CS	22	CD: 1 Carcinoma: 13 Adenoma: 5 Other: 3	1/22	21 : 1*	4.0^∗, #^	Extracorporeal	0/22	0/22	105^∗, #^	0/22	5/22 (wound infection: 1, ileus: 3, bleeding: 1)	2/22*	5^#^	n.s.

Champagne et al., 2011 [12]	CC	29	CD: 7 Carcinoma: 12 Adenoma: 4 Diverticulitis: 6	*/19	7 : 0	3.8*	Extracorporeal	1/7	0/7	134^∗, $^	0/29	5/29* (n.s.)	0/29	3.7*	n.s.

Chaudhary et al., 2011 [34]	CC	4	CD: 4	4/4	n.s.	n.s.	n.s.	n.s.	1/4	n.s.	n.s.	n.s.	n.s.	n.s.	n.s.

Gaujoux et al., 2011 [32]	CS	13	CD: 3 Adenoma: 5 Diverticulitis: 3 Other: 2	2/6	n.s.	3.7	Extracorporeal	0/2	0/2	155^§^	0/2	0/2	0/2	5^§^	n.s.

Gaujoux et al., 2012 [11]	CC	25	CD: 6 UC: 2 Carcinoma: 3 Adenoma: 8 Diverticulitis: 4 Other: 2	*/13	24 : 1^$^	n.s.	Extracorporeal	1/25*	0/6	130^∗#^	0/6	1/25* (acute urine retention)	0/6	6^#^	0/6

Gash et al., 2011 [17]	CS	20	CD: 4 UC: 3 Carcinoma: 8 Diverticulitis: 2 Other: 3	3/6	4 : 0	n.s.	n.s.	0/4	0/4	123^§^	0/4	5/20* (wound infection: 1, ileus: 2, anastomotic bleeding: 1, other: 1)	0/4	5.2^§^	1/20*

Geisler and Garrett, 2011 [8]	CS	102	CD: 14 UC: 51 Neoplasia: 23 Diverticulitis: 11 Other: 3	*/26	14 : 0	4.4*	Extracorporeal	18/102*	1/102*	77*	0/102*	39/102* (wound infection: 11, ileus: 12, pulmonary: 10, other: 6	0/26*	5.9*	0/26*

Karahasanoglu et al., 2011 [21]	CR	1	CD: 1	1/1	1 : 0	2.5	Intracorporeal	0/1	0/1	140	0/1	0/1	0/1	4	n.s.

Lee et al., 2011 [9]	CC	46	CD: 5 Neoplasia: 25 Diverticulitis: 16	*/24	n.s.	5.1*	Extracorporeal	2/24*	0/24*	122*	0/24	11/46* (wound infection: 4, anastomotic leak: 1, bleeding: 1, ileus: 1, other: 4	n.s.	4.6*	n.s.

Papaconstantinou et al., 2011 [15]	CC	29	CD: 2 Carcinoma: 15 Adenoma: 12	2/29	n.s.	4.5^§^	Extracorporeal	0/29*	0/29*	129^∗§^	0/29*	6/29* (wound infection: 5, anastomotic leak: 1)	1/29*	3.4^§^	4/29*

Ross et al., 2011 [10]	CS	39	CD: 5 Carcinoma: 15 Adenoma: 12 Divertculitis: 7	*/30	n.s.	4.2^§^	n.s.	3/39*	0/5	120*	0/5	3/39* (wound infection: 1, bleeding: 2)	0/5	4.4*	0/5

Scaringi et al., 2011 [22]	CR	1	CD: 1	1/1	1 : 0	n.s.	Extracorporeal	0/1	0/1	115	0/1	0/1	n.s.	5	n.s.

Stewart and Messaris, 2012 [27]	CS	41	CD: 7 UC: 6 Carcinoma: 11 Adenoma: 4 Diverticultis: 10 Other: 3	4/13	29 : 12*	3.2*	Extracorporeal	1/7	5/41*	178^∗$^	0/7	7/35* (anastomotic leak: 1, intraabd. abscess: 1, other: 5)	1/41	8.7^∗§^	5/35*

Vestweber et al., 2011 [20]	CS	200	CD: 21 UC: 16 Diverticulitis: 120 Other: 43	21/26	200 : 0*	n.s.	Extracorporeal	n.s.^$^	n.s.^§^	n.s.^§^	0/200*	n.s.^§^	n.s.^§^	9*	n.s.

Wolthuis et al., 2011 [23]	CC	14	CD: 6 Carcinoma: 5 Adenoma: 1 Diverticulitis: 2	5/10	14 : 0	5*	Extracorporeal	0/6	0/6	75^#∗^	0/5	0/5	1/5	7^#∗^	0/6

Champagne et al., 2012 [13]	CC	165	CD: 26 UC: 13 Carcinoma: 64 Adenoma: 41 Diverticulitis: 15 Other: 6	*/117	n.s.	n.s.	n.s.	14/165*	n.s.	135^∗§^	1/165*	42/165* (wound infection: 7, ileus: 15, delayed thermal injury: 1, bleeding: 1, cardiovascular: 4, other: 15	2/165*	4.3^∗§^	8/165*

Rijcken et al., 2012 [16]	CC	20	CD: 20	20/20	20 : 0	3.8^§^	Extracorporeal	0/20	1/20	137^§^	0/20	4/20 (wound infection: 2, anastomotic leak: 1, intraabd. abscess: 1)	1/20	9^§^	1/20

Stewart and Messaris, 2012 [35]	CS	6	CD: 6	6/6	n.s.	3.5^§^	Extracorporeal	1/6	0/6	160^§^	0/6	2/6 (wound infection: 1, intraabd. abscess: 1)	0/6	4.8^§^	0/6

Waters et al., 2012 [36]	CS	100	CD: 5 Carcinoma: 57 Adenoma: 5	5/100	95 : 5	3.5^§∗^	Extracorporeal	2/100*	4/100*	114^§∗^	1/100* (bleeding)	14/100* (wound infection: 4, ileus: 4, bleeding: 3, anastomotic leak/ abscess: 2, other: 1)	1/100*	5*	n.s.

*Not particularly specified for Crohn's disease

^$^Not specified for SPLS ileocecal resection-right hemicolectomy

^§^Mean value, ^#^median value

n.s.: not specified

CC: case-controlled study, CR: case report, CS: case series

CD: Crohn's disease, UC: ulcerative colitis.

**Table 2:** Perioperative results of SPLS subtotal colectomy in IBD: included studies.

Author, year	Study type	Total number of SPLS patients	Disease (*n*, all SPLS patients)	Subtotal colectomy IBD/ reconstruction	Elective: emergency	Final incision length (cm)	Ileorectal anastomosis	Additional trocars (*n*/cases)	Conversion to open surgery	Operative time (min)	Mortality (*n*/cases)	Morbidity (*n*/cases)	Reoperations (*n*/cases)	Hospital stay (*d*)	Read missions (*n*/cases)
Cahill et al., 2010 [28]	CS	3	CD: 1 UC: 2	3/End ileostomy: 3	0 : 3	2.0	—	0/3	0/3	206^§^	0/3	1/3 (ileus: 1)	1/3	5.3^§^	0/3

Chambers et al., 2011 [19]	CS	7	CD: 0 UC: 2 Carcinoma: 3 Diverticulitis: 1 Other: 1	1/End ileostomy: 1	n.s.	2.5	—	0/1	0/1	130	0/1	0/1	0/1	3	0/1

Fichera et al., 2011 [25]	CS	10	CD: 0 UC: 10	10/End ileostomy: 10	n.s.	n.s. (stoma site)	—	0/10	0/10	139^§^	0/10	n.s.	n.s.	5.1^§^	n.s.

Fichera et al., 2011 [37]	CC	10	CD: 0 UC: 10	10/End ileostomy: 10	n.s.	n.s. (stoma site)	—	0/10	0/10	139^§^	0/10	0/10	n.s.	5.1^§^	n.s.

Gaujoux et al., 2011 [32]	CS	13	CD: 3 Adenoma: 5 Diverticulitis: 3 Other: 2	1/End ileostomy: 1	n.s.	3.2^#^	—	0/1	0/1	150	0/1	0/1	n.s.	6	n.s.

Gaujoux et al., 2012 [11]	CC	25	CD: 6 UC: 2 Carcinoma: 3 Adenoma: 8 Diverticulitis: 4 Other: 2	2/End ileostomy: 2	24 : 1*	n.s.	—	0/2	0/2	130^#∗^	0/2	1/25* (acute urine retention)	0/2	6^#∗^	0/2

Gash et al., 2011 [17]	CS	20	CD: 4 UC: 3 Carcinoma: 8 Diverticulitis: 2 Other: 3	2/End ileostomy: 1 Ileo-rectal Anastomis: 1	2 : 0	n.s.	transanal	0/2	0/2	120^§^	0/2	5/20* (wound infection: 1, ileus: 2, anastomotic bleeding: 1, other: 1)	0/2	2^§^	1720*

Geisler and Garrett, 2011 [8]	CS	102	CD: 14 UC: 51 Neoplasia: 23 Diverticulitis: 11 Other: 3	19/End ileostomy: 19	19 : 0	n.s. (stoma site)	—	0/19	0/19	99*	0/19	39/102* (wound infection: 11, ileus: 12, pulmonary: 10, other: 6	0/19	5, 9*	0/19

Leblanc et al., 2011 [26]	CS	4	CD: 1 UC: 1	2/End ileostomy: 2	2 : 0	n.s. (stoma site)	—	0/2	0/2	162^§^	0/2	1/4* (ileus: 1)	0/2	4.5*	n.s.

Stewart and Messaris, 2012 [27]	CS	41	CD: 7 UC: 6 Carcinoma: 11 Adenoma: 4 Diverticultis: 10 Other: 3	6/End ileostomy: 6	29 : 12*	n.s. (stoma site)	—	0/6	1/6	155^§∗^	0/6	7/35* (anastomotic leak: 1, intraabd. abscess: 1, other: 5)	0/6	4.2*	0/6

Van den Boezem and Sietses, 2011 [30]	CS	50	CD: 0 UC: 4 Carcinoma: 31 Adenoma: 7 Diverticulitis: 8	4/End ileostomy: 2 Ileo-rectal anastomosis: 2	4 : 0	n.s.	transanal	4/50*	0/4	130^§∗^	0/4	10/50* (anastomotic leakage: 1, wound infections: 4, incisional hernia: 2, ileus: 2, other: 1)	0/4	6^#∗^	n.s.

Vestweber et al., 2011 [20]	CS	200	CD: 21 UC: 16 Diverticulitis: 120 Other: 43	10/End ileostomy: 10	10 : 0	n.s.	—	n.s.*	n.s.*	n.s.*	0/10	n.s.*	n.s.^§^	9*	n.s.

Champagne et al., 2012 [13]	CC	165	CD: 26 UC: 13 Carcinoma: 64 Adenoma: 41 Diverticulitis: 15 Other: 6	8/End ileostomy: 8	n.s.	n.s. (stoma site)	—	14/165*	n.s.	135^∗§^	1/165*	42/165* (wound infection: 7, ileus: 15, delayed thermal injury: 1, bleeding: 1, cardiovascular: 4, other: 15	2/165*	4.3^∗§^	8/165*

Fichera and Zoccoli, 2012 [24]	CS	9	UC: 9	9/End ileostomy: 9	n.s.	n.s. (stoma site)	—	0/9	0/9	142^§^	0/9	0/9	0/9	5.2^§^	n.s.

*Not particularly specified for subtotal colectomy

^§^Mean value, ^#^median value

n.s.: not specified

CC: case-controlled study, CR: case report, CS: case series

CD: Crohn's disease, UC: ulcerative colitis.

**Table 3:** Perioperative results of restorative proctocolectomy (IPAA) in ulcerative colitis: included studies.

Author, year	Study type	Total number of SPLS patients	Disease (*n*, all SPLS patients)	SPLS-IPAA	Elective: emergency	Final incisio*n* length (cm)	Anastomosis	Loop ileostomy	Additional trocars (*n*/cases)	Conversion to open surgery	Operative time (min)	Mortality (*n*/cases)	Morbidity (*n*/cases)	Reoperations (*n*/cases)	Hospital stay (*d*)	Read missions (*n*/cases)
Nagpal et al., 2010 [38]	CR	1	UC: 1	1	1 : 0	5.5	Stapler	1/1	1/1	0/1	256	0/1	n.s.	0/1	7	n.s.

Podolsky and Curcillo II, 2010 [4]	CS	13	UC: 1 Carcinoma: 8 Other: 4	1	n.s.	n.s.	n.s.	n.s.	n.s.	n.s.	300	0/13	3/13* (wound infection: 1, incisional hernia: 2)	n.s.	5	n.s.

Chambers et al., 2011 [19]	CS	7	CD: 0 UC: 2 Carcinoma: 3 Diverticulitis: 1 Other: 1	1	1 : 0	n.s.	n.s.	1/1	0/1	0/1	195	0/1	0/1	0/1	4	n.s.

Gash et al., 2011 [18]	CS	10	UC: 10	10	n.s.	2.5 (stoma site)	Stapler: 8 Hand-sewn: 2	9/10	0/10	0/10	185^#^	0/10	2/10 (other: 2)	0/10	3^#^	0/10

Gash et al., 2011 [17]	CS	20	CD: 4 UC: 3 Carcinoma: 8 Diverticulitis: 2 Other: 3	2	n.s.	n.s. (stoma site)	Stapler: 2	n.s.	1/2	0/2	177^§^	0/2	5/20* (wound infection: 1, ileus: 2, anastomotic bleeding: 1, other: 1)	0/2	3^§^	1/20*

Geisler and Garrett, 2011 [8]	CS	102	CD: 14 UC: 51 Neoplasia: 23 Diverticulitis: 11 Other: 3	20	20 : 0	n.s. (stoma site)	Stapler: 20	20/20	15/20	1/20	160	0/20	39/102* (wound infection: 11, ileus: 12, pulmonary: 10, other: 6	0/20	5, 9*	0/20

Geisler et al., 2011 [39]	CS	5	UC: 4 FAP: 1	5	4 : 0	n.s. (stoma site)	Stapler	4/4	0/4	0/4	175^§^	0/4	2/5* (ileus: 2)	0/4	4^#∗^	2/4

Leblanc et al., 2011 [26]	CS	4	CD: 1 UC: 2 FAP: 1	2	1 : 0	n.s. (stoma site)	Stapler	1/1	0/1	0/1	261*	0/1	0/1	0/1	4.5^#∗^	n.s.

Mattioli et al., 2011 [40]	CS	5	UC: 5	5	n.s.	n.s. (stoma site)	Stapler	5/5	n.s.	n.s.	n.s.	0/1	n.s.*	0/5	n.s.*	n.s.

Stewart and Messaris, 2012 [27]	CS	41	CD: 7 UC: 6 Carcinoma: 11 Adenoma: 4 Diverticultis: 10 Other: 3	2	n.s.	n.s. (stoma site)	Stapler	n.s.	0/2	0/2	155^$∗^	0/2	7/35* (anastomotic leak: 1, intraabd. abscess: 1, other: 5)	0/2	4.2*	0/2

Vestweber et al., 2011 [20]	CS	200	CD: 21 UC: 16 Diverticulitis: 120 Other: 43	6	6 : 0	n.s.	Hand-sewn	n.s.*	n.s.*	n.s.*	n.s.*	0/6	n.s.*	n.s.*	n.s*	n.s.*

Champagne et al., 2012 [13]	CC	165	CD: 26 UC: 13 Carcinoma: 64 Adenoma: 41 Diverticulitis: 15 Other: 6	8	n.s.	n.s. (stoma site)	n.s.	n.s.	14/165*	n.s.	135^∗§^	1/165*	42/165* (wound infection: 7, ileus: 15, delayed thermal injury: 1, bleeding: 1, cardiovascular: 4, other: 15	2/165*	4.3^∗§^	8/165*

*Not particularly specified for SPLS-IPAA in UC

^§^Mean value, ^#^median value

n.s.: not specified

CC: case-controlled study, CR: case report, CS: case series

CD: Crohn's disease, UC: ulcerative colitis, FAP: familial adenomatous polyposis

IPAA: Ileopouch-anal anastomosis.

## References

[1] Bucher P, Pugin F, Morel P (2008). Single port access laparoscopic right hemicolectomy. *International Journal of Colorectal Disease*.

[2] Remzi FH, Kirat HT, Kaouk JH, Geisler DP (2008). Single-port laparoscopy in colorectal surgery. *Colorectal Disease*.

[3] Leroy J, Cahill RA, Asakuma M, Dallemagne B, Marescaux J (2009). Single-access laparoscopic sigmoidectomy as definitive surgical management of prior diverticulitis in a human patient. *Archives of Surgery*.

[4] Podolsky ER, Curcillo PG (2010). Single port access (SPA) surgery-a 24-month experience. *Journal of Gastrointestinal Surgery*.

[5] Leblanc F, Champagne BJ, Augestad KM (2010). Single incision laparoscopic colectomy: technical aspects, feasibility, and expected benefits. *Diagnostic and Therapeutic Endoscopy*.

[6] Diana M, Dhumane P, Cahill RA, Mortensen N, Leroy J, Marescaux J (2011). Minimal invasive single-site surgery in colorectal procedures: current state of the art. *Journal of Minimal Access Surgery*.

[7] Makino T, Milsom JW, Lee SW (2012). Feasibility and safety of single-incision laparoscopic colectomy: a systematic review. *Annals of Surgery*.

[8] Geisler D, Garrett T (2011). Single incision laparoscopic colorectal surgery: a single surgeon experience of 102 consecutive cases. *Techniques in Coloproctology*.

[9] Lee SW, Milsom JW, Nash GM (2011). Single-incision versus multiport laparoscopic right and hand-assisted left colectomy: a case-matched comparison. *Diseases of the Colon and Rectum*.

[10] Ross H, Steele S, Whiteford M (2011). Early multi-institution experience with single-incision laparoscopic colectomy. *Diseases of the Colon and Rectum*.

[11] Gaujoux S, Maggiori L, Bretagnol F, Ferron M, Panis Y (2012). Safety, feasibility, and short-term outcomes of single port access colorectal surgery: a single institutional case-matched study. *Journal of Gastrointestinal Surgery*.

[12] Champagne BJ, Lee EC, Leblanc F, Stein SL, Delaney CP (2011). Single-incision vs straight laparoscopic segmental colectomy: a case-controlled study. *Diseases of the Colon and Rectum*.

[13] Champagne BJ, Papaconstantinou HT, Parmar SS (2012). Single-incision versus standard multiport laparoscopic colectomy: a multicenter, case-controlled comparison. *Annals of Surgery*.

[14] Kawahara H, Watanabe K, Ushigome T, Noaki R, Kobayashi S, Yanaga K (2010). Single-incision laparoscopic right colectomy for recurrent Crohn’s disease. *Hepato-Gastroenterology*.

[15] Papaconstantinou HT, Sharp N, Thomas JS (2011). Single-incision laparoscopic right colectomy: a case-matched comparison with standard laparoscopic and hand-assisted laparoscopic techniques. *Journal of the American College of Surgeons*.

[16] Rijcken E, Mennigen R, Argyris I, Senninger N, Bruewer M (2012). Single-incision laparoscopic surgery for ileocolic resection in Crohn’s disease. *Diseases of the Colon and Rectum*.

[17] Gash KJ, Goede AC, Chambers W, Greenslade GL, Dixon AR (2011). Laparoendoscopic single-site surgery is feasible in complex colorectal resections and could enable day case colectomy. *Surgical Endoscopy*.

[18] Gash KJ, Goede AC, Kaldowski B, Vestweber B, Dixon AR (2011). Single incision laparoscopic (SILS) restorative proctocolectomy with ileal pouch-anal anastomosis. *Surgical Endoscopy and Other Interventional Techniques*.

[19] Chambers WM, Bicsak M, Lamparelli M, Dixon AR (2011). Single-incision laparoscopic surgery (SILS) in complex colorectal surgery: a technique offering potential and not just cosmesis. *Colorectal Disease*.

[20] Vestweber B, Straub E, Kaldowski B (2011). Single-port colonic surgery: techniques and indications. *Der Chirurg*.

[21] Karahasanoglu T, Hamzaoglu I, Aytac E, Baca B (2011). Transvaginal assisted totally laparoscopic single-port right colectomy. *Journal of Laparoendoscopic and Advanced Surgical Techniques*.

[22] Scaringi S, Giudici F, Liscia G, Cenci C, Tonelli F (2011). Single-port laparoscopic access for Crohn’s disease complicated by enterocutaneous fistula. *Inflammatory Bowel Diseases*.

[23] Wolthuis AM, Penninckx F, Fieuws S, D’Hoore A (2012). Outcomes for case-matched single port colectomy are comparable with conventional laparoscopic colectomy. *Colorectal Disease*.

[24] Fichera A, Zoccali M (2012). Single-incision laparoscopic total abdominal colectomy for refractory ulcerative colitis. *Surgical Endoscopy*.

[25] Fichera A, Zoccali M, Felice C, Rubin DT (2011). Total abdominal colectomy for refractory ulcerative colitis. Surgical treatment in evolution. *Journal of Gastrointestinal Surgery*.

[26] Leblanc F, Makhija R, Champagne BJ, Delaney CP (2011). Single incision laparoscopic total colectomy and proctocolectomy for benign disease: initial experience. *Colorectal Disease*.

[27] Stewart DB, Messaris E (2012). Outcomes for consecutive patients undergoing single-site laparoscopic colorectal surgery. *Journal of Gastrointestinal Surgery*.

[28] Cahill RA, Lindsey I, Jones O, Guy R, Mortensen N, Cunningham C (2010). Single-port laparoscopic total colectomy for medically uncontrolled colitis. *Diseases of the Colon and Rectum*.

[29] Adair J, Gromski MA, Lim RB, Nagle D (2010). Single-incision laparoscopic right colectomy: experience with 17 consecutive cases and comparison with multiport laparoscopic right colectomy. *Diseases of the Colon and Rectum*.

[30] van den Boezem PB, Sietses C (2011). Single-incision laparoscopic colorectal surgery, experience with 50 consecutive cases. *Journal of Gastrointestinal Surgery*.

[31] Heeney A, O’Connor DB, Martin S, Winter DC (2010). Single-port access laparoscopic surgery for complex Crohn’s disease. *Inflammatory Bowel Diseases*.

[32] Gaujoux S, Bretagnol F, Ferron M, Panis Y (2011). Single-incision laparoscopic colonic surgery. *Colorectal Disease*.

[33] Keshava A, Young CJ, MacKenzie S (2010). Single-incision laparoscopic right hemicolectomy. *British Journal of Surgery*.

[34] Chaudhary B, Glancy D, Dixon AR (2011). Laparoscopic surgery for recurrent ileocolic Crohn’s disease is as safe and effective as primary resection. *Colorectal Disease*.

[35] Stewart DB, Messaris E (2012). Early experience with single-site laparoscopic surgery for complicated ileocolic Crohn’s disease at a tertiary-referral center. *Surgical Endoscopy*.

[36] Waters JA, Rapp BM, Guzman MJ (2012). Single-port laparoscopic right hemicolectomy: the first 100 resections. *Diseases of the Colon and Rectum*.

[37] Fichera A, Zoccali M, Gullo R (2011). Single incision (“scarless”) laparoscopic total abdominal colectomy with end ileostomy for ulcerative colitis. *Journal of Gastrointestinal Surgery*.

[38] Nagpal AP, Soni H, Haribhakti S (2010). Hybrid single-incision laparoscopic restorative proctocolectomy with ileal pouch anal anastomosis for ulcerative colitis. *Indian Journal of Surgery*.

[39] Geisler DP, Kirat HT, Remzi FH (2011). Single-port laparoscopic total proctocolectomy with ileal pouch-anal anastomosis: initial operative experience. *Surgical Endoscopy*.

[40] Mattioli G, Guida E, Pini-Prato A (2012). Technical considerations in children undergoing laparoscopic ileal-J-pouch anorectal anastomosis for ulcerative colitis. *Pediatric Surgery International*.

[41] Reissman P, Salky BA, Pfeifer J, Edye M, Jagelman DG, Wexner SD (1996). Laparoscopic surgery in the management of inflammatory bowel disease. *American Journal of Surgery*.

[42] Milsom JW, Hammerhofer KA, Böhm B, Marcello P, Elson P, Fazio VW (2001). Prospective, randomized trial comparing laparoscopic vs. conventional surgery for refractory ileocolic Crohn’s disease. *Diseases of the Colon and Rectum*.

[43] Moorthy K, Shaul T, Foley RJ (2004). Factors that predict conversion in patients undergoing laparoscopic surgery for Crohn’s disease. *American Journal of Surgery*.

[44] Okabayashi K, Hasegawa H, Watanabe M (2007). Indications for laparoscopic surgery for Crohn’s disease using the Vienna classification. *Colorectal Disease*.

[45] Soop M, Larson DW, Malireddy K, Cima RR, Young-Fadok TM, Dozois EJ (2009). Safety, feasibility, and short-term outcomes of laparoscopically assisted primary ileocolic resection for Crohn’s disease. *Surgical Endoscopy and Other Interventional Techniques*.

[46] Tan JJY, Tjandra JJ (2007). Laparoscopic surgery for Crohn’s disease: a meta-analysis. *Diseases of the Colon and Rectum*.

[47] Maartense S, Dunker MS, Slors JFM (2006). Laparoscopic-assisted versus open ileocolic resection for Crohn’s disease: a randomized trial. *Annals of Surgery*.

[48] Huscher CG, Mingoli A, Sgarzini G Standard laparoscopic versus single-incision laparoscopic colectomy for cancer.

